# 550. Bamlanivimab and Casirivimab/Imdevimab Treatment Outcomes: Results from a Large Healthcare System’s Structured Implementation Experience

**DOI:** 10.1093/ofid/ofab466.749

**Published:** 2021-12-04

**Authors:** Christopher Polk, Anna Jacobs, Mindy Sampson, Michael Leonard, Leigh Ann Medaris, Christopher Branner, Vineet Goel, Lisa Davidson

**Affiliations:** 1 Atrium Health, Charlotte, NC; 2 Carolinas Medical Center - Atrium Health, Charlotte, NC

## Abstract

**Background:**

Neutralizing antibody therapies targeting SARS-CoV-2 have been released for emergency use authorization by the FDA. Little is published on their real-world experience. In this retrospective study we share the results of our early experience on patient outcomes from use of these neutralizing antibodies within a large healthcare system.

**Methods:**

We retrospectively analyzed results of a healthcare system wide program to pro-actively identify and treat COVID-19 patients with neutralizing antibody therapy.

**Results:**

The 449 patients identified for SARS-CoV-2 neutralizing antibody therapy during the study period were retrospectively classified as falling in one of the three groups: untreated (199), bamlanivimab (87) and casirivimab/indevimab (125) treated patients (Table 1). Reasons identified patients were not treated most commonly were patient declined (n=74), unable to be contacted (n=36), out of treatment window (n=23), asymptomatic and feeling better (n=21) or did not have transportation (n=9). Bamlanivimab infusion did not reduce emergency room (ER) visits or hospitalization compared to untreated patient within 30-days of follow up (Table 2), and among all patients treated with antibody therapy only treatment with bamlanivimab and non-white race were predictors of need for hospitalization (Table 3). Casirivimab/indevimab did reduce subsequent ER visits or hospitalization within 30 days post-infusion when compared to the untreated group. However, patients treated with either antibody therapy had lower acuity of COVID-19 disease as reflected in need for intensive care unit (ICU) stay, mechanical ventilation or death (Table 2).

Table 1. Characteristics of infused vs uninfused patients

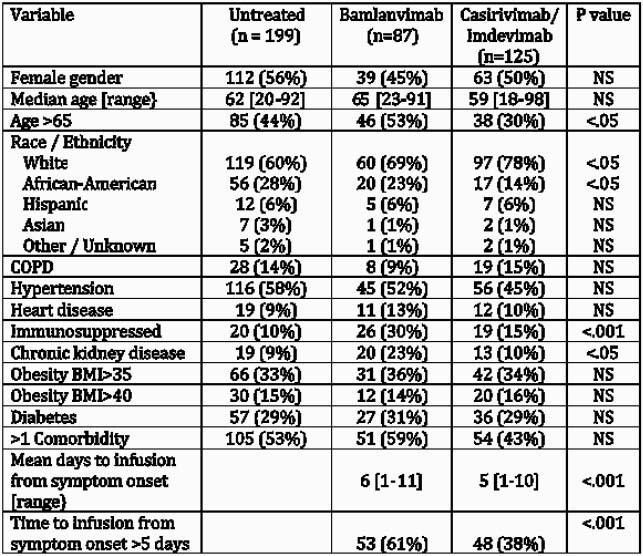

Table 2. Outcomes in treated vs untreated patients

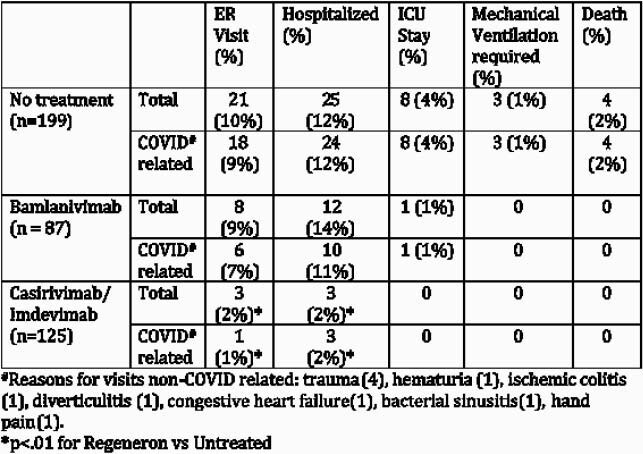

Table 3. Risk factors for ED visits or hospitalization in infused patients

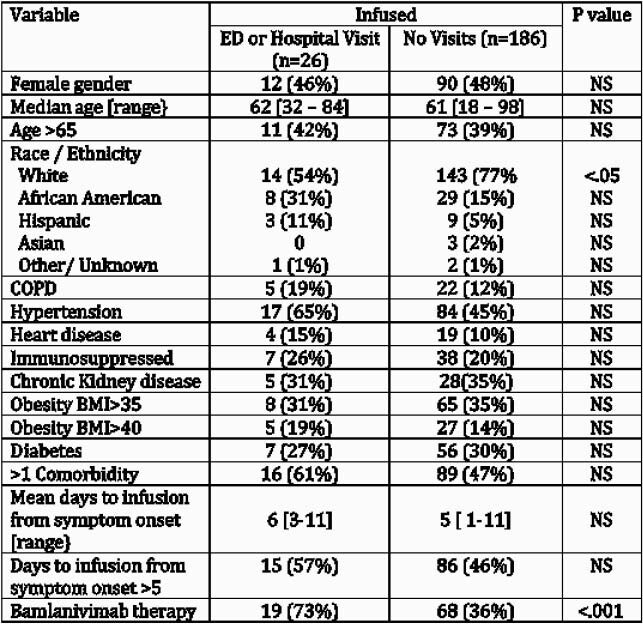

**Conclusion:**

Either neutralizing antibody therapy appears to markedly reduce acuity of COVID-19 disease even if patients do progress to requiring hospitalization. However, casirivimab/indevimab therapy also decreased ER visits and hospitalization suggesting better efficacy in our experience.

**Disclosures:**

**Christopher Polk, MD**, **Atea** (Research Grant or Support)**Gilead** (Advisor or Review Panel member, Research Grant or Support)**Humanigen** (Research Grant or Support)**Regeneron** (Research Grant or Support) **Mindy Sampson, MD**, **Regeneron** (Grant/Research Support)

